# Velocity-dependent slip weakening by the combined operation of pressure solution and foliation development

**DOI:** 10.1038/s41598-018-22889-3

**Published:** 2018-03-16

**Authors:** A. R. Niemeijer

**Affiliations:** Department of Earth Sciences, Utrecht University, HPT Laboratory, Utrecht, The Netherlands

## Abstract

Phyllosilicate-bearing faults are characterized by an anastomosing foliation with intervening hard clasts and are believed to be long-term weak structures. Here, I present results of sliding experiments on gouges of 80 wt% quartz and 20 wt% muscovite, sheared under hydrothermal conditions at constant velocity. The results show that significant strengthening occurs over a narrow range of sliding velocities (0.03–1 μm/s). At the lowest velocity investigated, weakness is achieved after a considerable sliding distance of over 20 mm with friction reaching a value of 0.3. Microstructural observations and the application of existing models point to the operation of frictional-viscous flow (FVF), through the serial operation of frictional sliding over a weak foliation and pressure solution of intervening clasts, resulting in low frictional strength and pronounced velocity-strengthening. At higher velocities, grain size reduction becomes dominant in a localized zone, which results in disruption of the foliation and the cessation of the FVF mechanism. In natural settings, earthquakes originating elsewhere on the fault would be rapidly arrested when encountering a foliated part of the fault deforming via FVF. Furthermore, pulses of elevated slip velocity would lead to grain size reduction which would destroy the foliation and cause a long-term strengthening of the fault.

## Introduction

Understanding the sliding strength of fault rocks over a wide range of loading conditions is important to better evaluate seismic hazard. Ground shaking is, among several other things, affected by the depth at which an earthquake nucleates, and by the total amount of coseismic slip^1^. The total amount of coseismic slip in turn depends on the ease with which an earthquake rupture can propagate, the elastic properties of the fault, its surroundings, and the stress distribution. However, the presence of phyllosilicates as well as the operation of fluid-assisted processes (e.g. pressure solution) might allow a fault to slip aseismically under much lower stress levels than those required for frictional processes and at much lower temperatures than those required to activate fully ductile flow (e.g.^2–5^, see^6^ for a review). This implies that such parts of the fault should allow for easy propagation of seismic slip, but only if strength remains low over the entire range of slip velocities and/or becomes even lower at coseismic slip velocities due to the operation of dynamic, thermally activated weakening mechanisms such as flash heating, thermal pressurization and melt lubrication (see^7–9^ for reviews). An additional important question is whether researchers can identify microstructures that are diagnostic for specific slip velocities and associated shear strain rates^9,10^.

## Previous work

**Figure 1:**
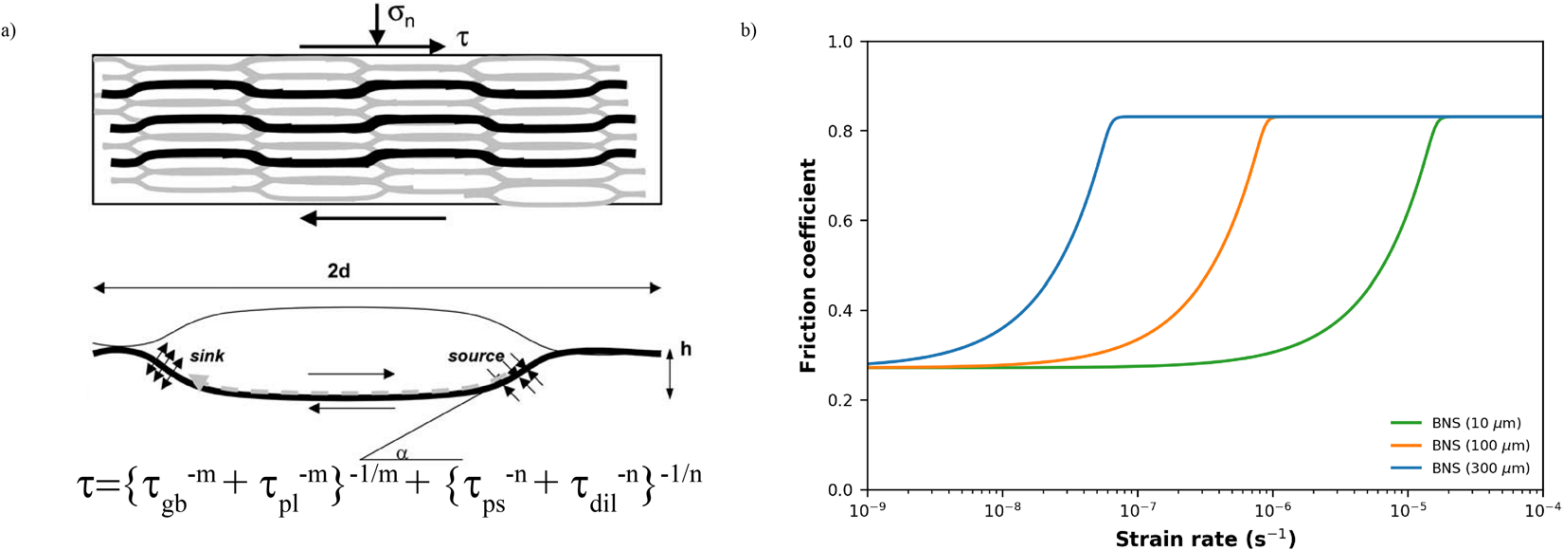
(**a**) Model geometry at steady state of the frictional-viscous flow (FVF) mechanism showing an anastomosing foliation (black lines) with intervening soluble grains (white). Deformation is accommodated through the serial processes of grain boundary friction (τ_gb_) or internal plastic deformation of the foliation (τ_pl_) with pressure solution (τ_ps_) or dilatation (τ_dil_). If the exponent m is large, the transition between the parallel processes (i.e. between τ_ps_ and τ_dil_ or between τ_gb_ and τ_pl_) is sharp, such as might be expected in a mixture with a single value grain size of the soluble phase (after Bos & Spiers, 2002 and Niemeijer & Spiers, 2005). (**b**)Model predictions for 80/20 wt% mixed quartz/muscovite fault zone, deforming at an effective normal stress of 100 MPa and a temperature of 300 °C. Note the large effect of grain size on the velocity at which velocity strengthening occurs.

There have been many experimental studies of frictional strength of both natural and analogue phyllosilicate-bearing fault gouges^11^. Typically, the presence of phyllosilicates leads to a lowering of the frictional strength of composite gouges, with the strength of the composite gouge approaching that of the phyllosilicate end member when the phyllosilicate content is 80 wt% or more^11,12^. The weakening is usually attributed to a switch in the load-bearing framework from the strong phase to the weak phase and is thus purely geometric. In contrast, a large amount of weakening was observed for analogue mixtures of halite and kaolinite^13,14^ and halite and muscovite^15^ with phyllosilicate contents as low as 10 wt%. In these studies, the weakening was attributed to both a development of the microstructure (a geometric component) as well as the operation of ductile deformation, specifically pressure solution. The experiments showed that weakness only occurred when a phyllosilicate foliation was present and pressure solution creep occurred quickly enough to accommodate the imposed shearing rate. The deformation mechanism was termed frictional-viscous flow (FVF) and involves the frictional sliding between the soluble (halite) grains and the anastomosing foliation with dissolution, diffusion and precipitation of the intervening soluble grains (Fig. 1), operating at near-zero porosity and producing a microstructure resembling a mylonite without the operation of dislocation creep^13,14,16^. This model and the experimental results are characterized by a normal stress-dependent frictional strength that is also strongly rate-dependent (Fig. 1). Additionally, the grain size of the soluble phase as well as the kinetics of dissolution, diffusion and precipitation play key roles in enabling FVF and controlling the strain rate at which it can operate. If pressure solution is too slow or the shearing rate is too high, other mechanisms such as dilatation and cataclasis operate to accommodate deformation and significant strengthening is expected. The operation of FVF was also included in the model of Den Hartog & Spiers^17^, based on experimental results under hydrothermal conditions on phyllosilicate-rich (65 wt%) quartz-gouge mixtures, but the dramatic weakening observed in the analogue experiments was not observed, probably due to the high sliding velocities employed (1–100 μm/s). In this contribution, I present results showing dramatic weakening in phyllosilicate-bearing quartz gouges under mid-crustal PT conditions and discuss similarities and differences between the hydrothermal experiments and those on the analogue mixtures at room temperature. Finally, I discuss the implications of these results on long-term fault strength and earthquake rupture propagation into creeping segments of large scale fault zones.

## Methods

Experiments were performed in a hydrothermal ring shear apparatus that has been described in detail elsewhere (Figures S1 and S2^18^). The sample material consists of mixtures with 80 wt% quartz (Sil-co-sil 49, U.S. Silica company) and 20 wt% muscovite (crushed and sieved at d < 50 μm single crystals of high purity muscovite single crystals, grade V4, SPI supplies). In order to activate FVF, it is necessary to choose the conditions such that pressure solution is active at an appreciable rate. This requirement was met using a temperature of 500 °C, an effective normal stress of 120 MPa and a fluid pressure of 80 MPa, based on results from previous compaction experiments^19^. Samples were sheared at constant velocity ranging from 0.03 to 300 μm/s (bulk shear strain rates of ~3.10^−5^ to 3.10^−1^ s^−1^) for 30 mm displacement. At the end of the experiment, shear stress was removed, followed by cooling within ~30 minutes and removal of fluid pressure and normal stress. For experiments performed at low velocity (*v* < 1 μm/s, the ring-shaped sample could be extracted from the piston assembly in one piece. At higher velocities, the samples typically broke along angled fractures, resulting in several 10 to 15 mm long fragments. These fragments were impregnated with epoxy resin (Araldite 20/20) under vacuum and thin sections were prepared oriented approximately parallel to the shear direction. Microstructural analysis was done using a regular polarizing microscope equipped with a Leica camera, a JEOL JXA-8600 electron microprobe, a FEI Nova Nanolab scanning electron microscope equipped with a three-channel cathodoluminescence detector and a FEI Talos F200X transmission electron microscope.

## Results

**Figure 2:**
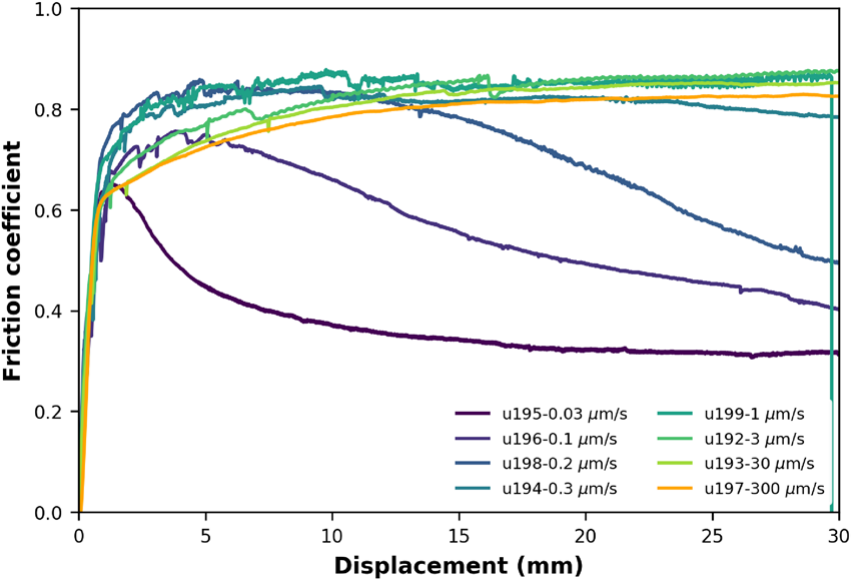
Evolution of friction (= shear stress/effective normal stress, ignoring cohesion) with displacement for all constant velocity experiments conducted using a mixture of 80 wt% quartz (Sil-co-Sil 49) and 20 wt% muscovite (SPI Grade V4 muscovite). Thermal conditions were somewhat unstable, especially initially in experiment u199, causing fluctuations in temperature of up to 30 °C, which affected fluid pressure and shear stress. Temperature was within 2–5 °C of the desired temperature in all other experiments.

Figure 2 shows the evolution of friction (= shear stress/effective normal stress, ignoring cohesion) as a function of displacement for all constant velocity experiments reported here. For all sliding velocities greater than 0.3 μm/s, the friction coefficient (friction) continuously increases during initial sliding, reaching µ ~ 0.8 at displacements of 3–5 mm. Continued shearing results in little change in friction, apart from artifacts caused by thermal fluctuations due to unstable temperature control. Samples sheared at velocities of 0.3 μm/s and below reach a similar or slightly lower maximum friction, but subsequently weaken with ongoing displacement. The displacement at which weakening is first observed decreases systematically with decreasing velocity, while at the same time the total amount of weakening increases (Fig. 2).

**Figure 3:**
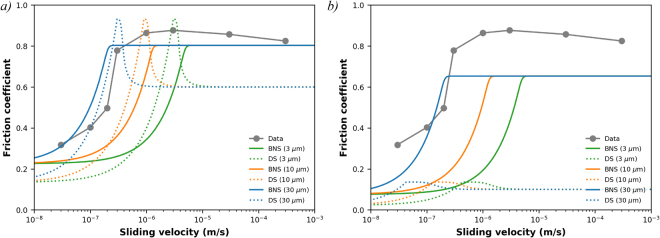
(**a**) Steady state friction determined at 30 mm displacement as a function of sliding velocity for all constant velocity experiments. Curves show results of model calculations using the model by Bos & Spiers (2002) modified by Niemeijer & Spiers (2005) and the model by den Hartog and Spiers (2014). More details about the parameters used can be found in the supplementary material. (**b**) Same as a) but now showing model results using a grain boundary friction of 0.1.

The friction coefficient at a displacement of 30 mm (or a shear strain of ~35–45, see Table 1) is plotted as a function of sliding velocity in Fig. 3 for all experiments. Friction has a peak at a sliding velocity of 3 μm/s with weakening at both faster and slower velocities, with the latter being particularly pronounced. The slope of this plot can be interpreted as a measure of the rate-and-state friction (RSF) parameter (*a* − *b*), which, at steady state is defined as^20^:

1 $$(a-b)=\frac{{\rm{\Delta }}\mu }{\mathrm{ln}\,{\rm{\Delta }}V}$$

**Table 1:** List of key mechanical data of all experiments reported.

Experiment	v (μm/s)	x (mm)	γ (−)	μ_initial_	h_0_ (mm)	μ_final_	h_final_ (mm)
u192	3	30.4	44.6	0.59	0.796	0.88	0.630
u193	30	30.4	36.2	0.62	0.905	0.86	0.769
u194	0.3	30.4	40.7	0.61	0.873	0.79	0.679
u195	0.03	30.9	38.3	0.64	0.950	0.32	0.730
u196	0.1	30.4	42.8	0.58	0.840	0.40	0.672
u197	300	30.2	40.1	0.60	0.798	0.83	0.693
u198	0.2	30.4	47.9	0.65	0.764	0.49	0.588
u199	1.0	29.7	38.5	0.68	0.860	0.86	0.725
u401	300-0.6	61.2	78.9	0.70	0.854	0.83	0.693
u403	300-0.1	63.3	80.2	0.73	0.911	0.53	0.696

All experiments were done at a temperature of 500 ºC, an effective normal stress of 120 MPa and a fluid pressure of 80 MPa. *x* denotes load-point displacement, γ is engineering shear strain (= displacement/instantaneous layer thickness), μ_initial_ is the friction at γ = 1, h_0_ is the layer thickness at the start of shearing, μ_final_ and h_final_ are the friction and the layer thickness at the end of shearing, respectively.

The difference in friction between 0.2 and 0.3 μm/s is about 0.3, which would give a *(a − b)*-value of 0.74, at least an order of magnitude larger than typical values obtained at room temperature^20^. Note here that the displacement is much larger than those typically employed in velocity-stepping experiments (30 mm vs. 0.5 mm^21^).

**Figure 4:**
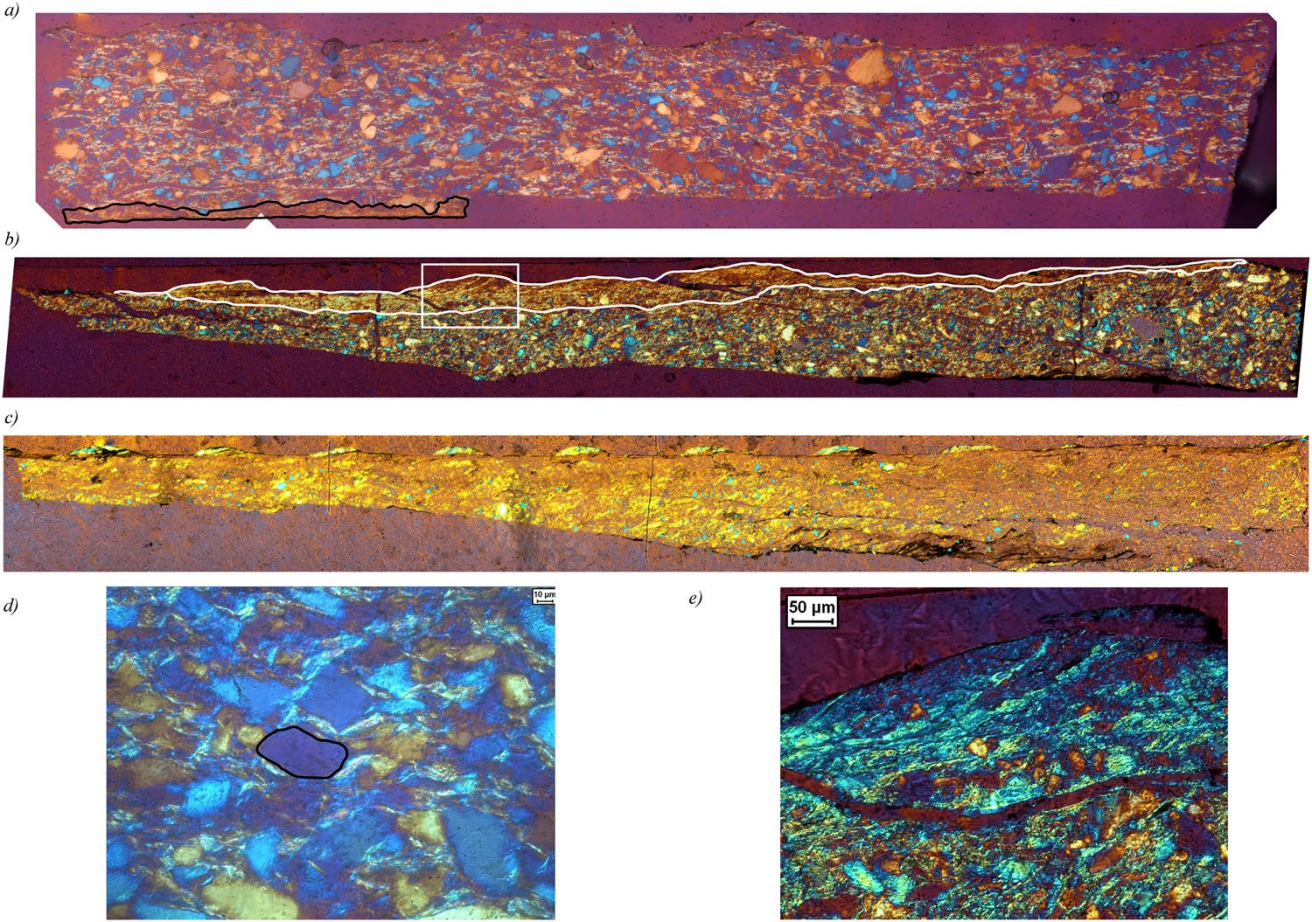
Mosaics of light microscope images of sheared samples, taken with the crossed nickels and the sensitive tint (gypsum) plate inserted. (**a**) Sample u195 (v = 0.03 μm/s. Shear sense is top-to-the-right. Thickness of the layer is ~600 μm, length of the section is 3.7 mm. A part of a zone with reduced grain size below the resolution of the light microscope (~1 μm) and with a uniform extinction color is visible along the and is outlined with black lines. (**b**) Sample u194 (v = 0.3 μm/s). Shear sense is top-to-the-right. Thickness of the layer is 730 μm, length of the section is ~7.1 mm. A zone with reduced grain size below the resolution of the light microscope (~1 μm) and with a uniform extinction color is visible along the boundary and is outlined with white lines. The white box indicates the location of the close-up, shown in e). (**c**) Sample u197 (v = 300 μm/s. Shear sense is top-to-the-right. Thickness of the layer is 790 μm, length of the section is ~8.4 mm. (**d**) Detail of the microstructure of sample u195, showing an slightly elongated quartz grain (outlined in black), resembling the idealized model microstructure of FVF shown in Fig. 1. (**e**) Detail of Fig. 4b), showing the boundary zone.

**Figure 5:**
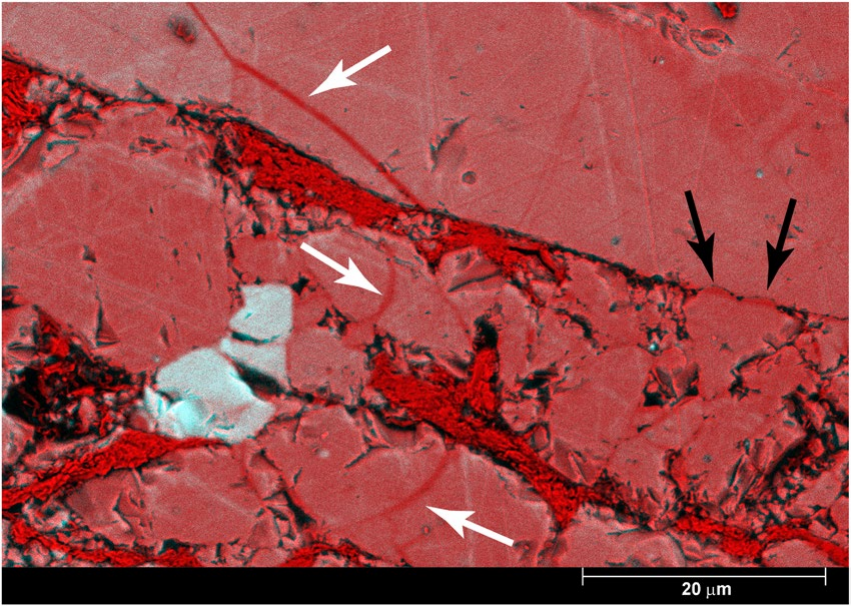
RGB composite image of BSE and CL images of of sample u195, sheared at 0.03 μm/s with the BSE signal in the red channel and the CL signal in the blue and green channels. Areas with an absence of CL signal are clearly visible in red and can be interpreted to be newly precipitated material in healed cracks (indicated with white arrows) or quartz overgrowths (black arrows), which are clear evidence for the operation of solution-transfer processes. Also note the three grains in white, indicating that these are grains with a strong CL signal, and the tight grain contacts they share with their neighbours to the left and to the right, which is another indication of the operation of solution-transfer processes. Muscovite grains can be seen in red as well, since they emit no CL signal.

**Figure 6:**
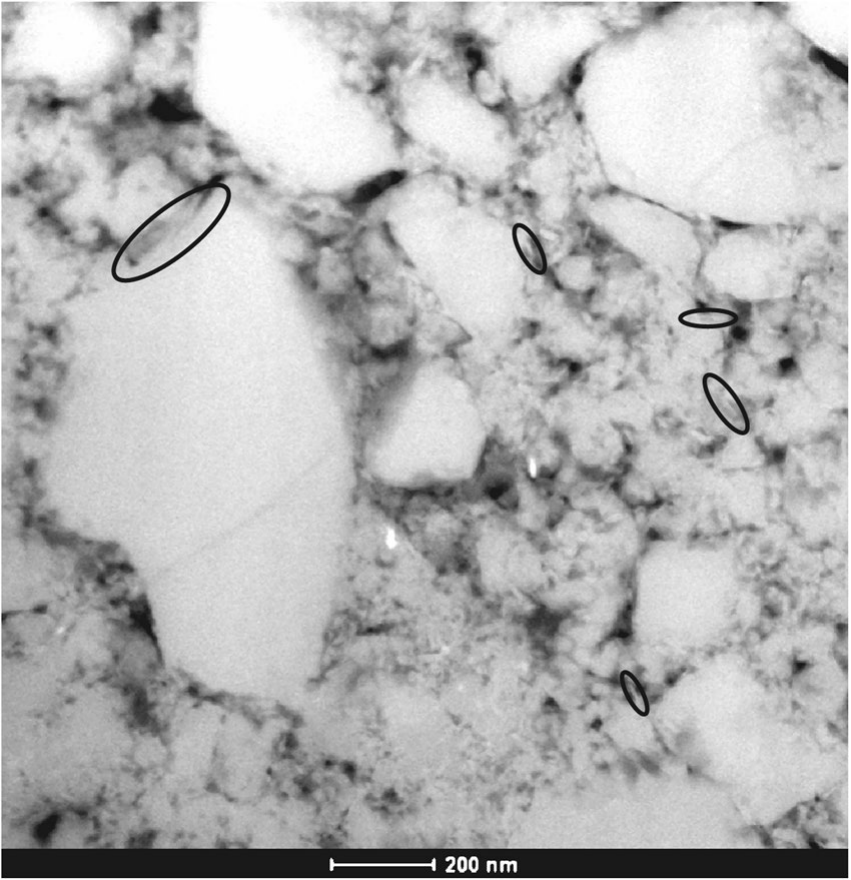
Transmission electron microscopy image in high angle angular dark-field (HAADF) mode of sample u197, sheared at 300 μm/s. Note the grain size of the quartz is smaller than ~500 nm, with grains as small as 10s of nm. Platy shaped objects (indicated by black ellipses) are interpreted to be muscovite grains and have sizes in the 10s of nm range.

All samples reveal the presence of a (sometimes partly recovered during extraction) zone of reduced grain size along the boundary of the sample, defining a boundary (B) shear^22^ (Fig. 4). Observations in the light microscope, with crossed nicols and the gypsum plate inserted, reveal a uniform extinction of the boundary shear, indicating the presence of a crystallographic preferred orientation (CPO^10^). The sample sheared at the highest velocity shows only a few grains larger than ~5 μm and almost the entire thin section displays a uniform extinction (Fig. 4c). At the lowest velocity, most muscovite grains are located in between quartz contacts and appear to form an anastomosing foliation (Fig. 4a and Supplementary Figure 9). High-resolution chemical mapping using the microprobe reveals that the quartz grains in the spectator regions are smaller and more angular when sheared at higher velocity (compare Supplementary Figures 8 and 9). Additionally, the porosity of the matrix appears to increase with increasing sliding velocity. Grains in the matrix of the sample deformed at the lowest velocity are often rounded and elongated, but angular grains are present as well (Supplementary Figure S6). Cathodoluminescence imaging of this sample (u195) reveals the presence of healed fractures and overgrowths indicating the operation of solution-transfer processes (Fig. 5). TEM imaging on sample u197, sheared at 300 μm/s, shows that the quartz grains in the boundary shears range in size between 10 nm to several micrometers, although even smaller grains might be present (Fig. 6). Also note in Fig. 6 the presence of grains with a platy appearance, which I interpret to be muscovite grains. EDS analysis of these and similar grains is part of ongoing work.

## Discussion – origin of slip weakening

**Figure 7:**
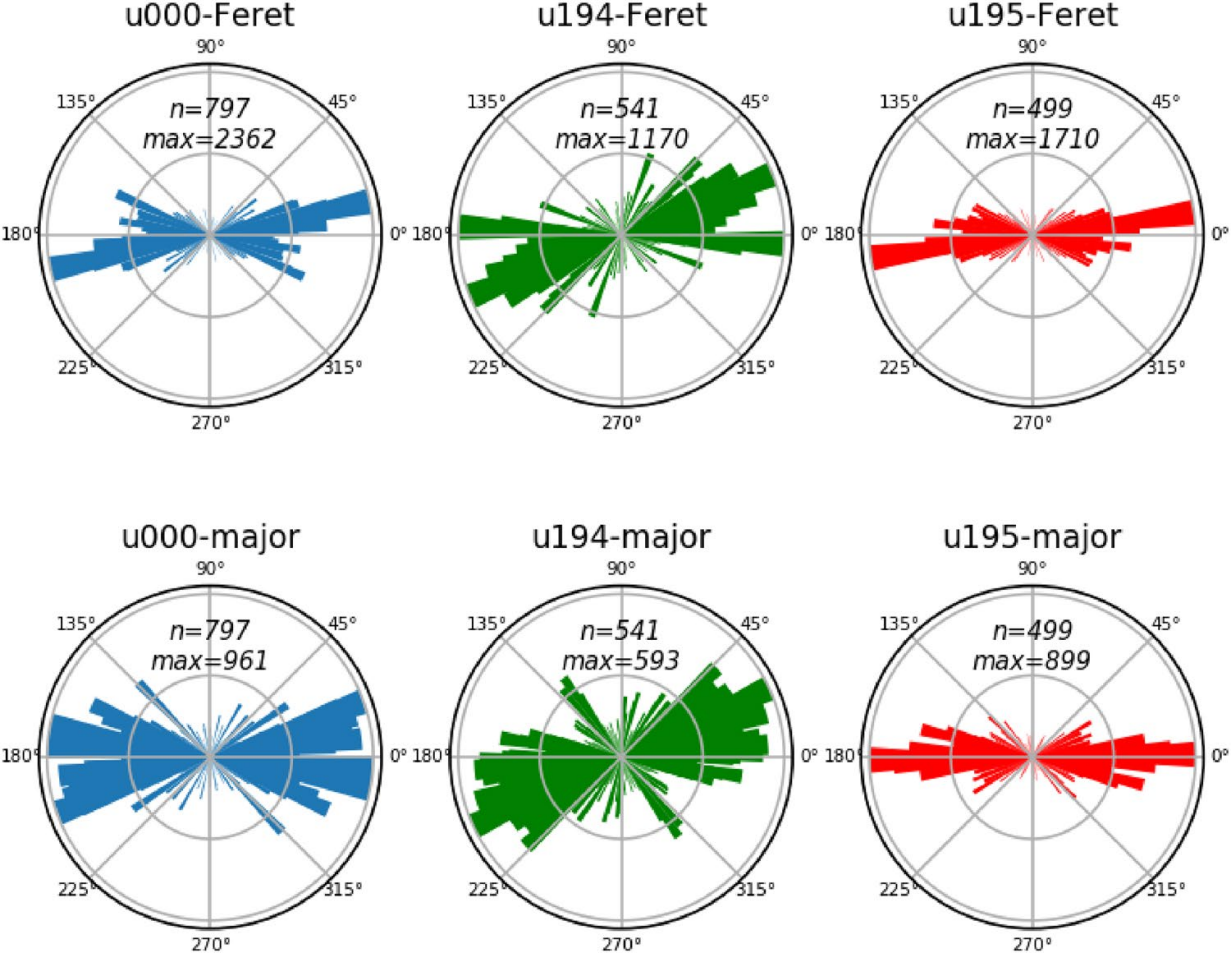
Rose diagrams of the distribution of orientations of the relative lengths of the Feret diameters and of the long axes of fitted ellipses of the connected regions of the muscovite phase with a 100 pixel (= 50 μm) minimum size for samples u000 (no shear), u194 (v = 0.3 μm/s) and u195 (v = 0.03 μm/s). Shear sense for is top-to-the-east. Note the strong (shape) preferred orientation observed in sample u195 (v = 0.03 μm/s).

Similar to previous results obtained on analogue materials^13–15^, frictional strength of the mixture of quartz with 20 wt% muscovite drops dramatically with decreasing sliding velocity. The results presented here are in good agreement with a frictional-viscous flow (FVF) mechanism (see Fig. 1). Significantly, friction evolves over a large displacement or strain, indicating that some geometrical evolution in the microstructure is needed to establish weakness. Additionally, the sliding velocities imposed in the current experiments are too fast to allow for the operation of dislocation creep in the quartz grains. The latter is demonstrated using the parameters of the flow law of Hirth *et al*.^23^ for dislocation creep in quartz, which gives a flow stress of ~1138 MPa for a strain rate of 10^−5^ s^−1^. Because this calculated flow stress far exceeds the shear stress recorded in the experiments, the quartz-muscovite mixture is inferred to deform by frictional-viscous flow at the lowest velocity investigated. Both the original model by Bos, Spiers and Niemeijer (BNS-model^15,16^) as well as a later version of the model by Den Hartog and Spiers (DS-model^17^ reproduce the trend in the experimental data (Fig. 3). Note that both models assume a single value grain size, whereas a distributed grain size would lead to a larger range of velocities over which the weakening occurs^15^. An important, but poorly constrained parameter for both models is the grain boundary friction (μ_gb_), which controls sliding of the quartz grains along the muscovite foliation. Values that were used are 0.3 in the BNS-model, on the basis of room temperature results on simulated brine-saturated gouges of muscovite, and 0.6 in the DS-model, on the basis of high temperature results of muscovite gouges^24^. Both these values are much higher than frictional sliding of muscovite single sheets over each other, both at room temperature (μ = 0.172^25^) and at 600 °C (μ = 0.08, Supplementary material 5). This indicates that frictional sliding of bulk muscovite gouges not only involves slip over the weak (001) planes, but must involve some rotation, buckling, bending or breaking of muscovite grains, particularly at the high velocities of 10 μm/s employed in den Hartog *et al*.^24^. In contrast, in the mixed gouges of muscovite and quartz, given enough strain and low enough velocity, the muscovite grains are aligned, and deformation is most likely accommodated by sliding over the (frictionally) weak (001) planes. This would suggest that a value of 0.1 is more appropriate for the grain boundary friction in the model, which would significantly change the model results (Fig. 3b). A sharp increase in strength with velocity remains, particularly in the BNS model, but the absolute values of friction are underestimated. However, the microphysical BNS model assumes a steady state microstructure consisting of an anastomosing foliation in which all deformation can be accommodated by the serial processes of sliding over the foliation and pressure solution of the intervening grains (Fig. 1). In the analogue mixture experiments, shear strains of 1000 were needed before the microstructure reached a steady state, resembling the assumed model geometry^14^. To quantify the existence and extent of a through-going anastomosing foliation as assumed in the model, image analyses using ImageJ were performed (see Supplemental material 3). Connected regions of muscovite with an area of 50 μm^2^ were identified based on microprobe chemical analyses of thin sections. The identified regions were subsequently fitted with ellipses and the lengths and orientations of the major axes measured. In addition, the Feret diameters, which is the longest possible distance between 2 pixels within the connected region, and their orientations were measured. Figure 7 shows the Rose diagrams of these analyses, plotting the relative cumulative lengths of each parameter in bins of 1°. Figure 7a shows that the starting microstructure displays a fabric with most of the muscovite grains oriented with their long axes perpendicular to the compression direction, which is at 90°. The distribution of orientations of the long axes changes slightly in sample u195, deformed at the lowest sliding velocity (0.03 μm/s Fig. 7c), whereas the dominant orientation is at an angle of 22 ° to the shear direction in experiment u194, deformed an order of magnitude faster (0.3 μm/s Fig. 7b). Initial shear loading in a granular aggregate, accompanied by compaction, causes a rotation of the internal stress field. In monomineralic gouges this typically leads to the formation of R_1_ shears at an angle of ~15 ° or 165 ° on the Rose diagram of experiment u194^22,26^. In a clay- or other phyllosilicate-rich gouges, P shears oriented in the opposite direction to R1 shears might form, which seems most consistent with the dominant orientation in u194 and perhaps also u195 (at least for the Feret diameter). However, the orientation of both P- and R_1_-shears are expected to become smaller with ongoing displacement, ultimately forming a boundary parallel or Y shear^26^. This shallowing of the localized shear bands is typically completed by a shear strain of ~5 or so^26^, which might suggest that most of the deformation in u194 is accommodated in the zone of enhanced grain size reduction along the boundary with the rigid loading piston. Returning to sample u195, for FVF to occur, it is necessary that the muscovite grains become connected and form a through-going network, which explains the long displacement over which weakening occurs (~20 mm). The fact that the final friction is still well above that of sliding of muscovite-muscovite (001) planes (μ = 0.3 vs. μ = 0.08) suggests that more displacement is needed to form a truly through-going foliated network. At the same time, the maximum weakness occurs and stabilizes by a displacement of approximately 20 mm. Three possible scenarios are indicated by these data: (1) the foliation is still developing at the end of the experiment, but the weakening is too small to distinguish, (2) the friction of quartz-muscovite sliding is larger than the friction of muscovite-muscovite sliding, or (3) the combination of quartz grain size and volumetric proportion of muscovite grains does not allow a continuous network of muscovite to be formed. To evaluate whether scenario (1) is correct, an experiment to very high shear strain would be needed, which is not practical. Scenario (2) would require experiments specifically aimed at measuring the friction of the quartz-muscovite interface. Scenario (3) might play a role in the experiments, since the microstructural analyses indicate that the single longest connected region does not exceed 300 μm, which is less than 20% of the entire length analysed. This suggests that some quartz-quartz contacts must have been actively sliding, contributing to the strength.

**Figure 8:**
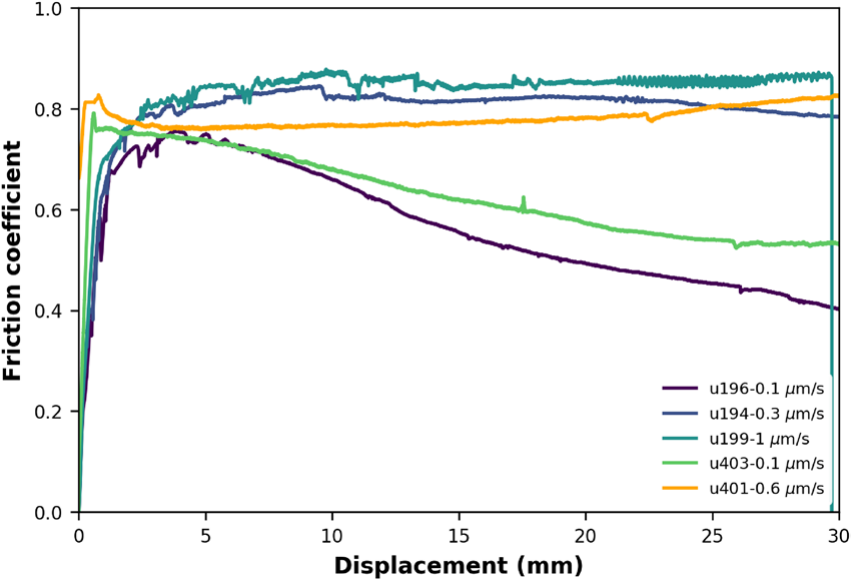
Evolution of friction with displacement for two additional experiments, u401 and u403, both initially sheared at 300 μm/s for 30 mm, before velocity was decreased to 0.1 and 0.6 μm/s. Only the slow shearing is shown here for an easy comparison with constant velocity experiments. Both experiments show that friction remains above the level of the experiments sheared only at low velocity, indicating that the grain size reduction induced by the fast shearing does not lead to more weakening, which was expected from the model predictions (for the effect of grain size on predicted frictional strength, see Fig. 3).

The microphysical models predict a pronounced effect of grain size on the critical velocity at which significant velocity-strengthening occurs (Figs 1 and 4). A smaller grain size should allow weakening to occur up to a higher velocity. In order to test the latter hypothesis, I performed two additional experiments in which the sample was sheared at the highest velocity first to produce a very fine-grained microstructure (Fig. 4c), which was subsequently sheared slowly. The experiments show considerably less weakening which occurs over a larger displacement than in the “standard” experiments (compare with u196, Fig. 8). An explanation for this apparent conflict with the model might be found in the amount of muscovite that is needed to cover all quartz contacts. When the quartz grain size is smaller, the total grain surface area for a fixed volume is larger. Assuming that the proportion of surface area that is in contact stays roughly the same, this indicates that more muscovite is needed to cover all quartz-quartz contacts. At the same time, a reduction in muscovite grain size would have the opposite effect. Calculations demonstrating these effects are shown in the Supplementary material and demonstrate that the quartz grain size effect dominates over the muscovite grain size effect. Note that here I assume a preferred location for the muscovite grains at the quartz contacts, which is fundamentally different from mixing formulations based on grain packing of two phases^11,27^. In the latter type of mixing formulations, quartz grain size should not play a role in the critical fraction of weak phase needed to form an interconnected network. In fact, in the experiments of Tembe *et al*.^11^ and similar works^12,28^ friction is observed to increase with increasing displacement for binary mixtures of phyllosilicates and quartz, which was attributed to ongoing cataclasis of the quartz “obstacles”. Additionally, weakening observed due to the addition of a phyllosilicate phase required at least 25 wt% and the residual strength was intermediate between the end-members, contrary to the results presented here. This is because in those room temperature studies, the hard quartz phase was unable to accommodate shear by a “viscous” mechanism, such as pressure solution, and thus had to dilate and/or break, contributing significantly to the overall strength due to the work done by both processes.

An additional factor to be considered in the delayed onset of weakening of sample u403 (Fig. 8) is the reduction of muscovite grain size. Small grains of muscovite would rotate more easily than large grains, disrupting the formation of a through-going foliation. Note that the zone of grain size reduction was excluded from the microstructural analyses presented in Fig. 7, because the grains could not be resolved with the 0.5 μm resolution used in the microprobe analyses. (see Supplemental Material 4). A reduction in muscovite grain size also has a chemical effect, whereby the exposure of fresh surfaces will allow for an almost instantaneous reaction of an uptake of H^+^ from the fluid and a release of K^+^^29^. This will change the local equilibrium composition, allowing for dissolution of the muscovite and precipitation of K-feldspar. Whether K-feldspar was produced in experiments u401 and u403 is currently being evaluated.

All the sheared samples show one or two boundary parallel zones of highly comminuted grains that increase in width with increasing sliding velocities, suggesting that shear deformation is accommodated within these layers^30,31^. However, this cannot be the case in the present experiments, at least at low velocity, for three reasons. First, if all shear had been accommodated by the boundary shear at the lowest sliding velocity, the strength would be controlled by this zone. The experiment that was sheared at high velocity first produced a microstructure that is similar, at least on a macroscopic scale, to that present within the localized zone at low velocity (compare Fig. 4a,b and c). However, subsequent shearing at low velocity did not lead to an immediate drop in friction, and additional displacement was required before the sample started to weaken (Fig. 8, compare experiment u403 with u196). Second, the width of the boundary shear increases with increasing sliding velocity (Fig. 4). Because strain rate is linearly related to the thickness of the actively deforming zone, an increasing width with increasing velocity would keep strain rate more or less constant and bulk frictional strength should not change. Third, if shearing was strongly localized, then the weakening with displacement must be associated with this localization. However, weakening at the one but lowest velocity (experiment u196, 0.1 μm/s) requires a much larger displacement than at the lowest velocity (experiment u195, 0.03 μm/s) and the magnitude of weakening is less even though the localized shear zone is wider (∆μ = ∼0.3 vs ∆μ = ∼0.4). Therefore, deformation at the lowest sliding velocity was not localized within the narrow boundary zone of (enhanced) grain size reduction and strength was controlled by the operation of frictional-viscous flow within the bulk of the gouge layer. Overall, the observed strengthening with increasing sliding velocity over a narrow range fits the trends predicted by the microphysical models, suggesting that within this velocity range (*v* = 0.1–1.0 μm/s, at least some of the deformation was also accommodated by frictional-viscous flow within the bulk of the sample.

A crystallographic preferred orientation (CPO) is typically associated with the operation of dislocation creep in natural samples. In the samples studied here, the CPO appears to be caused by quartz, although this has not been confirmed conclusively, because of the difficulty of measuring a CPO in very fine-grained material. However, deformation of quartz via dislocation creep at 500 °C at shear strain rates similar to those applied in the experiments (10^−5^–10^−2^ s^−1^) would require shear stresses well exceeding the 80–100 MPa measured. It is therefore unlikely that dislocation creep controls the overall deformation and development of the CPO, although its activity at highly stressed grain contacts can not be excluded. Power & Tullis^10^ observed uniform extinction of a thin zone of very fine-grained quartz in an outcrop of the Stillwater Fault zone (Nevada, U.S.A.) and suggested that this was caused by faster growth and dissolution of quartz in the direction of the c-axis, i.e. by pressure solution. Toy *et al*.^32^ proposed that the formation of a CPO in aggregates of nano-size particles could be due to a minimization of surface energy by particle rotation during annealing after their formation. Both mechanisms are plausible for the experimental conditions explored here, although preliminary TEM observations do not indicate significant proportions of healed grain contacts, which would be expected after annealing.

### Implications

The microphysical models predict that FVF is an efficient deformation mechanism for quartzo-feldspathic materials at relatively low temperatures of 250–300 °C^15–17^ (see Fig. 1). Considering that there are numerous examples from field studies showing microstructures with evidence for pressure solution and foliation development^33–36^, this suggests that mature, phyllosilicate-bearing faults can slide at shear stress levels well below those expected for friction in the range of Byerlee’s rule (see also^6^), even with phyllosilicate-contents as low as 20 wt%, as long as the phyllosilicates form an interconnected network and the rate of pressure solution in the other phase is comparable to or faster than the bulk shear strain rate. An important, relatively poorly constrained factor, is the actual sliding behaviour under hydrothermal conditions of the numerous phyllosilicates potentially present in faults. Flow laws for biotite and muscovite^37–39^ suggest that both of these phyllosilicates should deform by dislocation glide at shear stresses below those typically measured for phyllosilicate gouges^40,41^, at least at low shear strain rates. At the same time, it might be more instructive to consider the sliding strength of the soluble mineral over the phyllosilicate (e.g. quartz over muscovite) as the controlling parameter, but little is known about that process.

The long-term weakness of a foliated fault core controlled by FVF would not allow for any earthquakes to nucleate. However, along-strike or down-dip variations in fault mineralogy, fluid chemistry or grain size could lead to large variations in frictional strength. For example, a section of the fault that lacks phyllosilicates will be strong and potentially seismogenic. Any earthquakes nucleating in such a section would, however, be rapidly arrested in an adjacent foliated section of the fault due to the large negative stress drop associated with strong velocity strengthening. At the same time, pulses of high slip velocity would cause significant grain size reduction in the foliated section which would destroy the existing foliation, strengthen the fault, and potentially prime it for further rupture propagation in a subsequent earthquake. This experimental evidence and theoretical considerations suggest a strong coupling of the evolution of fault strength with physico-chemical changes, which needs to be constrained to better understand the long-term strength of faults and along-strike and down-dip extent of earthquake rupture propagation.

### Data Availability

Mechanical and microstructural data are available through reference^42^.

## Electronic supplementary material

Supplementary Dataset
