# Supplementary material for: Velocity-dependent slip weakening by the combined operation of pressure solution and foliation development
Source: Sci Rep. 2018 Mar 16;8:4724. doi: 10.1038/s41598-018-22889-3 (PMC5856847; doi:10.1038/s41598-018-22889-3)
Supplement: Supplementary file 1 — Supplementary Dataset [file 41598_2018_22889_MOESM1_ESM.docx]

Supplementary material to

*“Velocity-dependent slip weakening by the combined operation of pressure solution and foliation development”*

*A. R. Niemeijer*

This material consists of 7 parts:

1. Pictures of experimental apparatus and pistons used
2. Evolution of layer thickness with displacement for all constant velocity experiments
3. Calculation of the amount of muscovite needed to coat all possible quartz grain contacts as a function of quartz grain size and muscovite aspect ratio
4. Description of the quantitative microstructural analysis
5. Supplemental microstructures
6. Experimental results of frictional sliding experiments on stacks of single crystals of muscovite at 600 ºC
7. Values of the parameter input for the microphysical BNS and DS models

*Supplemental section 1: Pictures and diagrams of the experimental apparatus and pistons used*

*a)*

*b)*

**Figure S1.**

1. Photograph of the hydrothermal pressure vessel inside the Instron loading frame
2. Schematic drawing of the components of the pressure vessel (from Niemeijer et al, 2008).

**

*a)*

*
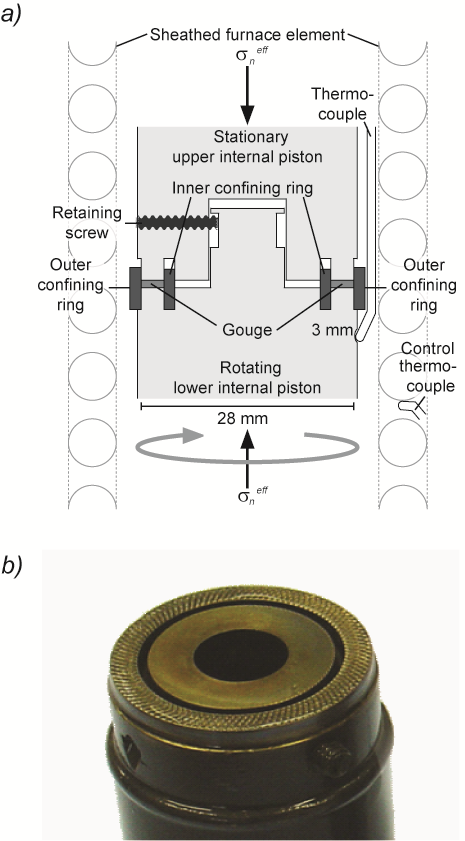
*

**Figure S2.**

1. Schematic drawing of the piston assembly (after Niemeijer et al., 2008)
2. Picture of the roughened surface of the top (non-rotating) piston.

*Supplemental section 3: Evolution of layer thickness*


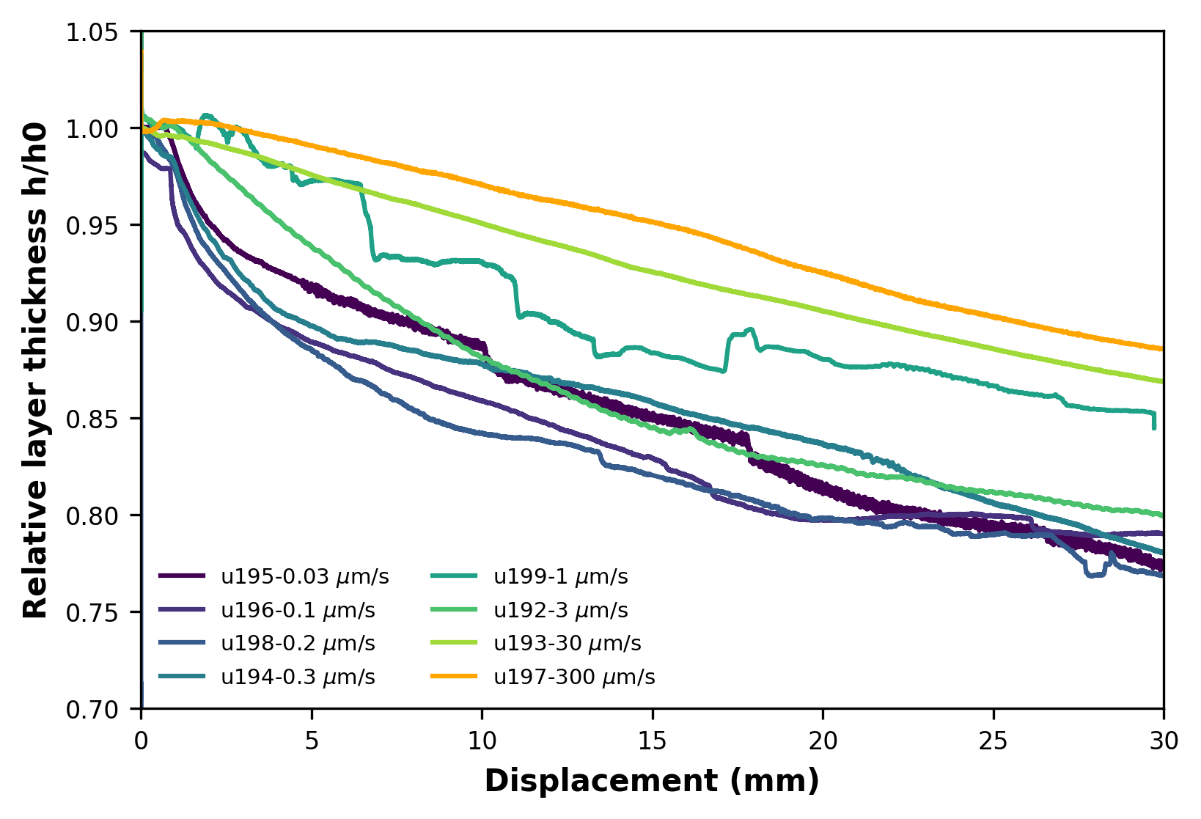


**Figure S3.**

Evolution of relative layer thickness as a function of displacement for all experiments at constant velocity. Layer thickness is normalized with respect to the layer thickness at the start of sliding (see also Table 1).

Supplemental section 3: Potential effect of grain size reduction on phyllosilicate foliation development

As a starting point, I assume that there is a mixture of two phases, in which one phase consist of sheet silicates which can bend and/or kink easily. In order for all imposed shear displacement to be accommodated by sliding in and over the sheet silicates, they need to form a continuous through-going network, without any contacts of the second phase.
Let’s assume that the second phase consists of spherical particles with a radius r. The surface area per particle is given by:

$A=4\pi r^{2}$ and the volume is $V=\frac{4}{3}\pi r^{3}$

At the same time, let’s assume that the sheet silicate particles are box-shaped with an aspect ratio b, given by l/w and a thickness t. The volume of the particle is then given by:

$V=l*w*t=b*w^{2}*t$ and the surface area of its 001 plane is A=l*w or b*w^2^

Let’s assume a proportion of phyllosilicates, Y and a porosity φ. For a total volume of 1, we have

$${Nr}^{qtz}=\left( 1-\phi\right)*\left( 1-Y \right)/\frac{4}{3}\pi r^{3}$$

The total surface area of quartz grains is then:

${Atot}^{qtz}=\left( 1-\phi\right)*\frac{\left( 1-Y \right)}{\frac{4}{3}\pi r^{3}}*4\pi r^{2}=\left( 1-\phi\right)*\frac{\left( 1-Y \right)}{\frac{1}{3}r}$

The number of phyllosicilate grains in the volume is given by:

$${Nr}^{qtz}=\left( 1-\phi\right)*Y/b*w^{2}*t$$

Which have a total surface area of:

${Atot}^{ph}=\left( 1-\phi\right)*\frac{Y}{b*w^{2}*t}*b*w^{2}=\left( 1-\phi\right)*\frac{Y}{t}$

The percentage of quartz surface area covered by phyllosilicates is thus:

$$A_{covered}=\frac{\left( 1-\phi\right)*\frac{Y}{t}*\frac{1}{3}r}{\left( 1-\phi\right)*(1-Y)}=\frac{\frac{Y}{t}*\frac{1}{3}r}{(1-Y)}$$

Or the volume proportion of phyllosilicates needed is given by:

$$\left( 1-Y \right)*A=\frac{Y}{t}*\frac{1}{3}r=> \frac{1-Y}{Y}=\frac{r}{3t*A}$$

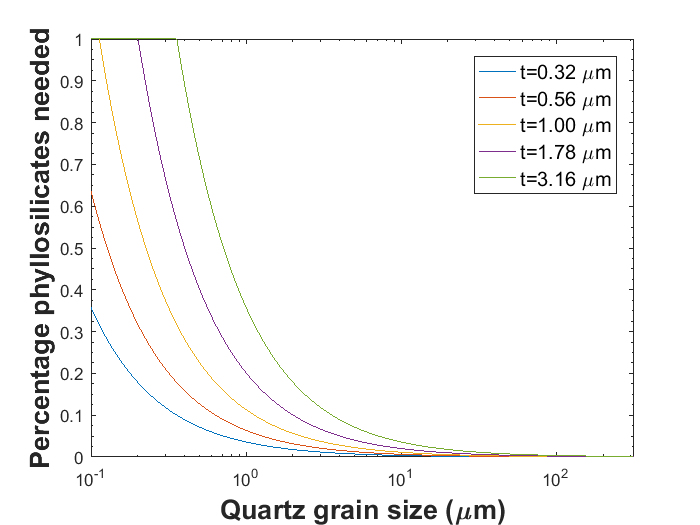

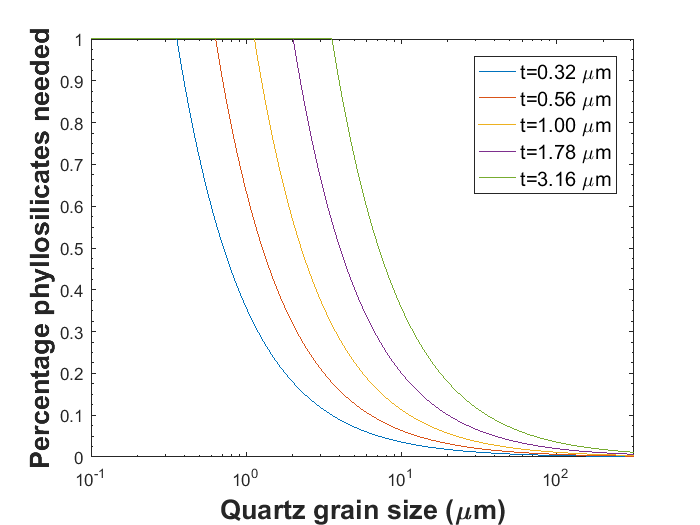


**Figure S4:**

*Calculated proportion of phyllosilicates needed to cover 1% of the surface area (left) and 10% of the quartz surface area (right). The relative amount of surface area of quartz grains will depend on the porosity and grain shape. Lines are shown for different thicknesses of the muscovite grains.*

Supplemental section 4: Analysis of presence and interconnectivity of a foliation

We analysed the microstructures of three different samples, u000, 194 and 195 using the microprobe. A region of a length about double the width of the samples was analysed using Wavelength Dispersive Spectroscopy with a resolution of 0.5 micron per pixel and a dwell time of 120 ms. From the resulting chemical maps, the signals of Aluminium and Potassium were logically added, i.e. only pixels with a non-zero value for both aluminium and potassium were used. This results in the images shown in Supplementary Figure 4, basically showing the location of the muscovite grains. Thresholding and counting the relative proportion of white pixels leads to respectively a 21, 24 and 23 area percent of muscovite, which is close to the expected value based on a composition with 20 wt% muscovite (Supplementary Table 1). From the binary images, we identified the connected regions with at least 100 pixels, or 50 μm. From these, the particle shapes were inferred by closing and filling of the holes, resulting in the images shown in Supplementary Figure 5. Analysis of the particle shapes and arrangement was done using the analyze particles feature in ImageJ as well as the PAROR software of Heilbronner and Barret (2014). It was found that PAROR analysis gave incorrect results for the unedited binary images, whereas the results of the ImageJ particle analysis were little affected by the close and fill holes operations. However, those operations did result in a much higher number of pixels identified (see Supplementary Table). Therefore, I decided to report the results of the analysis on the connected regions of the unedited binary image only.

**Supplementary Table 1.**

*Results of digital image analysis of the starting microstructure (u000) and two experimental microstructures (u194, v=0.3 mm/s and u195, v=0.03 mm/s). The numbers refer to the different methods used to determine the parameters, i.e. 1) refers to the major axis of the fitted ellipses using ImageJ 2) refers to the Feret diameter and 3) refers to the major axis of the fitted ellipses using PAROR.*

*Angle refers to the angle of the longest cumulative length, Cum. L is the cumulative length in pixels, Max. L is the maximum length of one element, with its angle in parenthesis, n/a is not available and desp. means despeckled.*

| **Sample/ method** | **Count**  **%pixel** | **Angle 1** | **Angle 2** | **Angle 3** | **Cum. L.**  **1** | **Cum. L.**  **2** | **Cum. L.**  **3** | **Max L.**  **1** | **Max L.**  **2** | **Max L. 3** |
| --- | --- | --- | --- | --- | --- | --- | --- | --- | --- | --- |
| u195 Al&K *binary* | 796  20.44 | 179 | 172 | n/a | 899 | 1711 | n/a | 197 | 329 | n/a |
| u195 Al&K *shapes* | 499  18.27 | 003 | 004 | 004 | 1254 | 1229 | 1900 | 441 | 596 | 596 |
| u195 Al&K *desp. shapes* | 610  10.21 | 179 | 172 | 176 | 904 | 947 | 1376 | 252 | 338 | 338 |
| u000 Al&K  *binary* | 797  19.36 | 169 | 192 | 45 | 962 | 2362 | 307 | 335  (162) | 886  (171) | 149  (36) |
| u000 Al&K  *shapes* | 416  34.49 | 007 | 002 | 022 | 1495 | 2273 | 1437 | 951  (007) | 1967  (002) | 1152  (014) |
| u000 Al&K  *desp. binary* | 1162  19.42 | 004 | 006 | n/a | 1215 | 1966 | n/a | 288  (168) | 473  (027) | n/a |
| u000 Al&K  *desp. shapes* | 797  23.48 | 178 | 175 | 022 | 1395 | 1906 | 1815 | 495  (168) | 696  (165) | 696  (165) |
| u194 Al&K  *shapes* | 541  22.78 | 025 | 022 | 178 | 594 | 1170 | 1067 | 355  (025) | 651  (022) | 651  (022) |
| u194 Al&K  *desp. shapes* | 659  12.92 | 028 | 030 | 029 | 631 | 734 | 843 | 315  (079) | 482  (066) | 482  (066) |

*
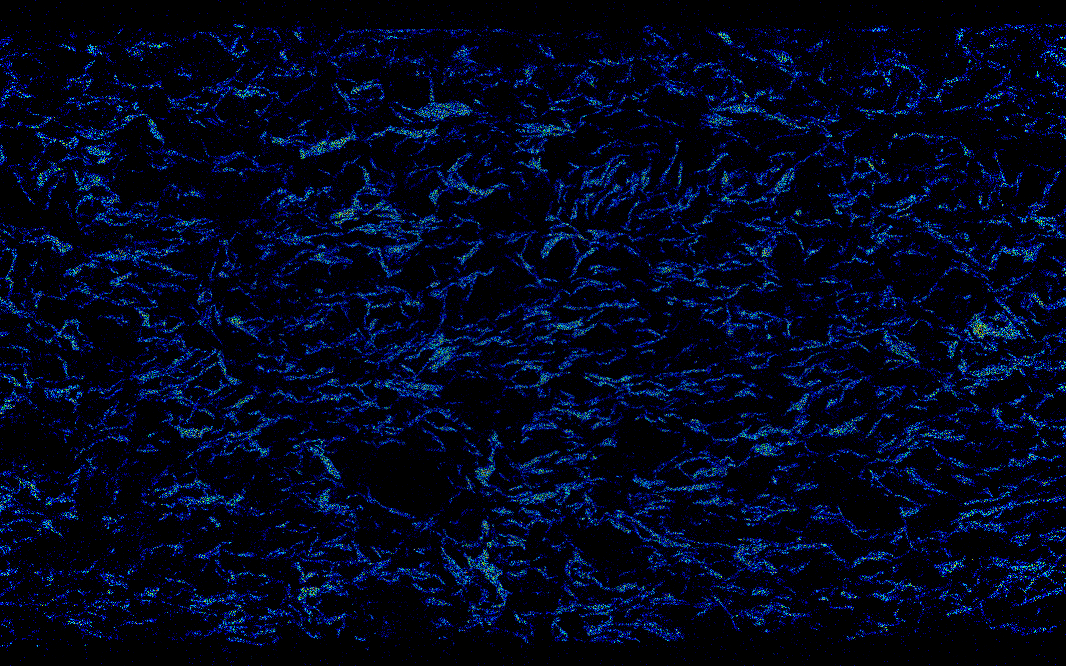
*

*
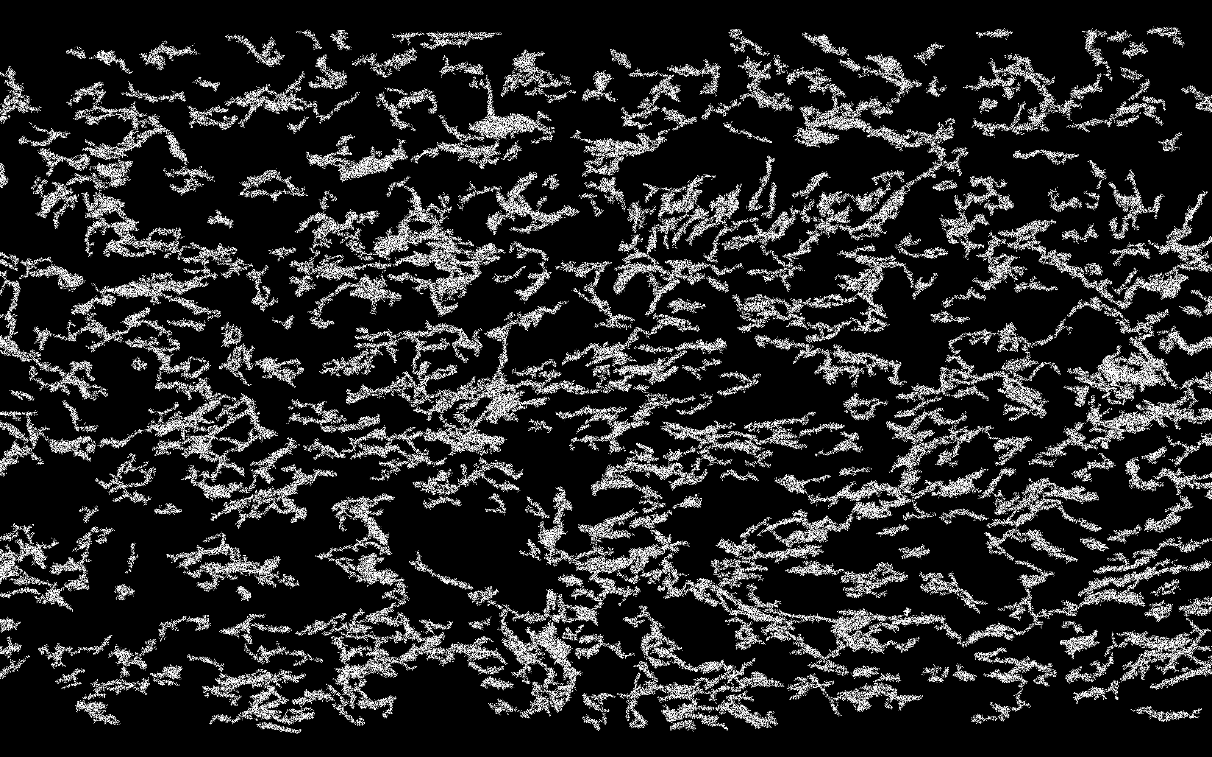
*

*Figure S5:*

*a) Combined map of Al & K signals of sample u000. Only pixels wtih a value for both have a value of Al+K. Map size is 2000x3200 pixels (=1000x1600 mm).*

*b) Connected regions of a minimum size of 100 pixels in image a).*

*
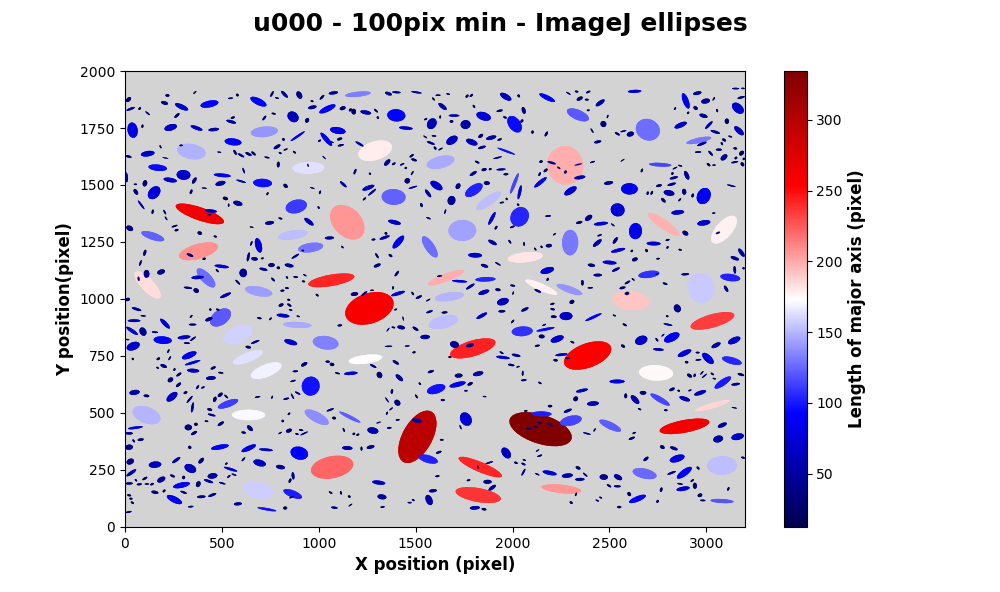

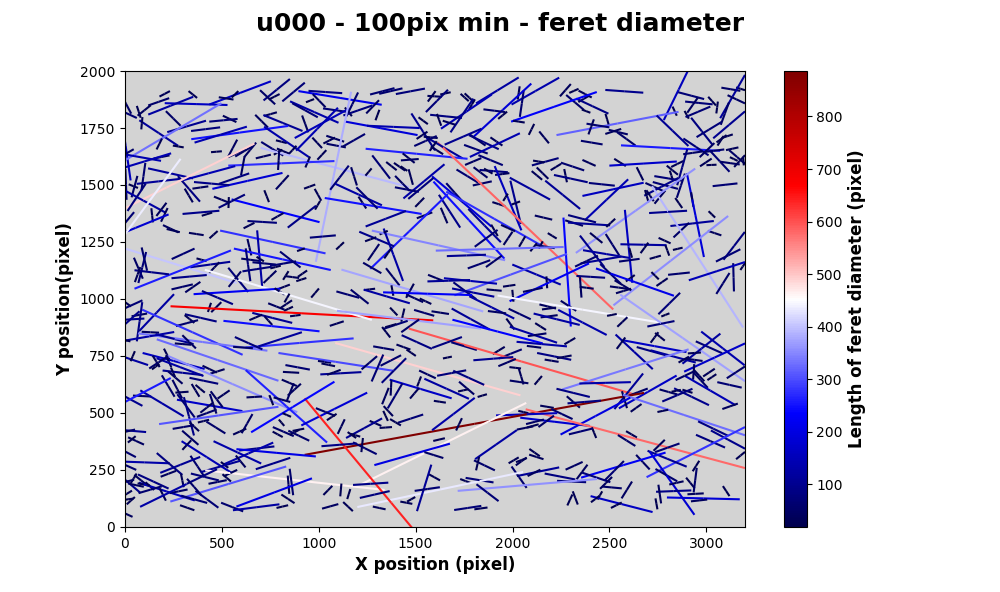
*

*Figure S6:*

*a) Plot of locations and lengths of the major axis of the fitted ellipses to the image in Figure 4b. This information is obtained using the particle analysis of ImageJ and used to construct the Rose diagrams shown in the Figure 7 of the main text.*

*b) Same as a) but for the Feret diameter.*

*Supplemental section 4): additional microstructures*

*
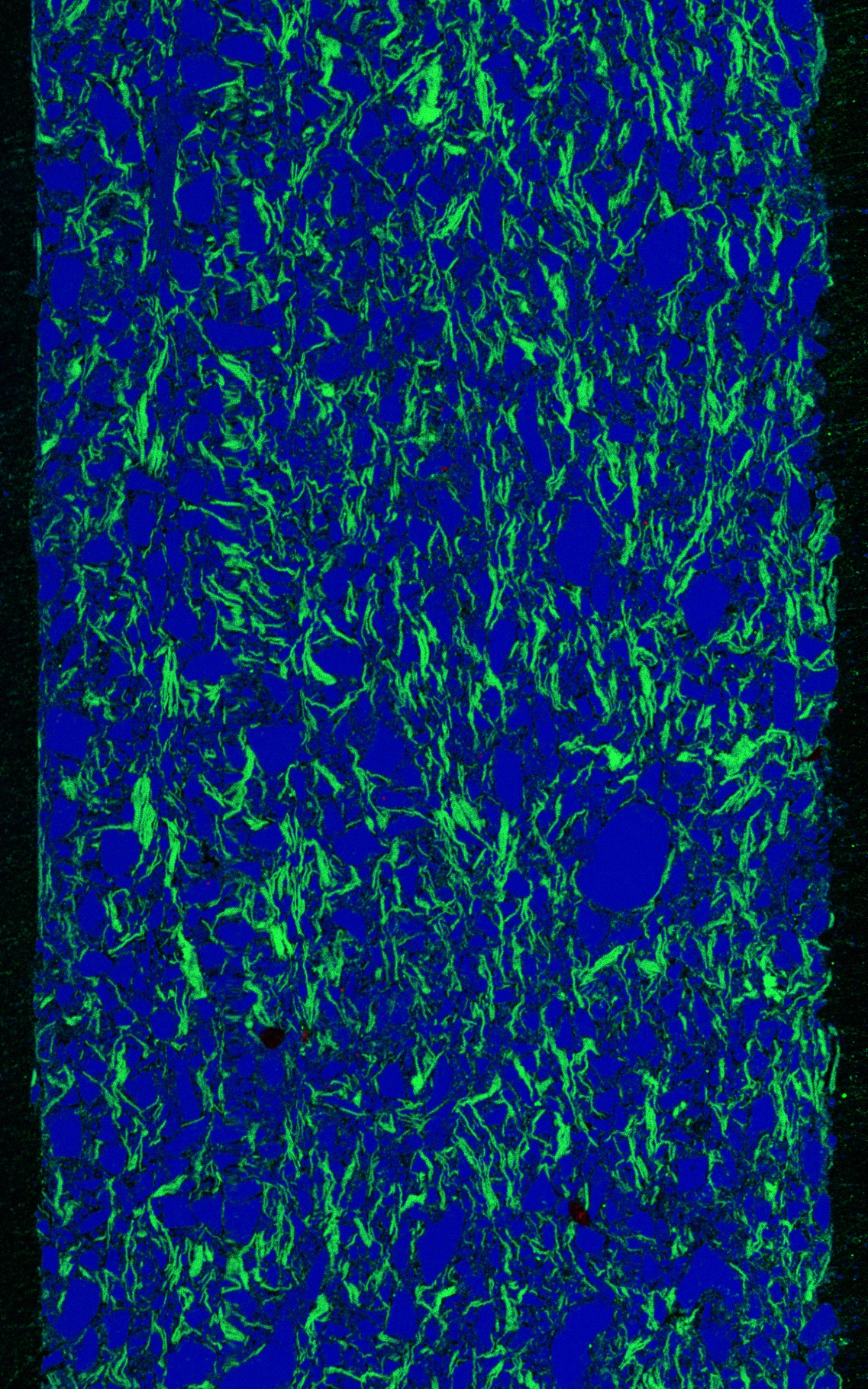
*

*Figure S7: RGB composite map of Si (blue), Al (green) and K (red) of sample u000, the starting microstructure. Data used in analysis of orientation of muscovite connected regions. Resolution is 0.5 μm per pixel, map is 2000x3200 pixels.*

*
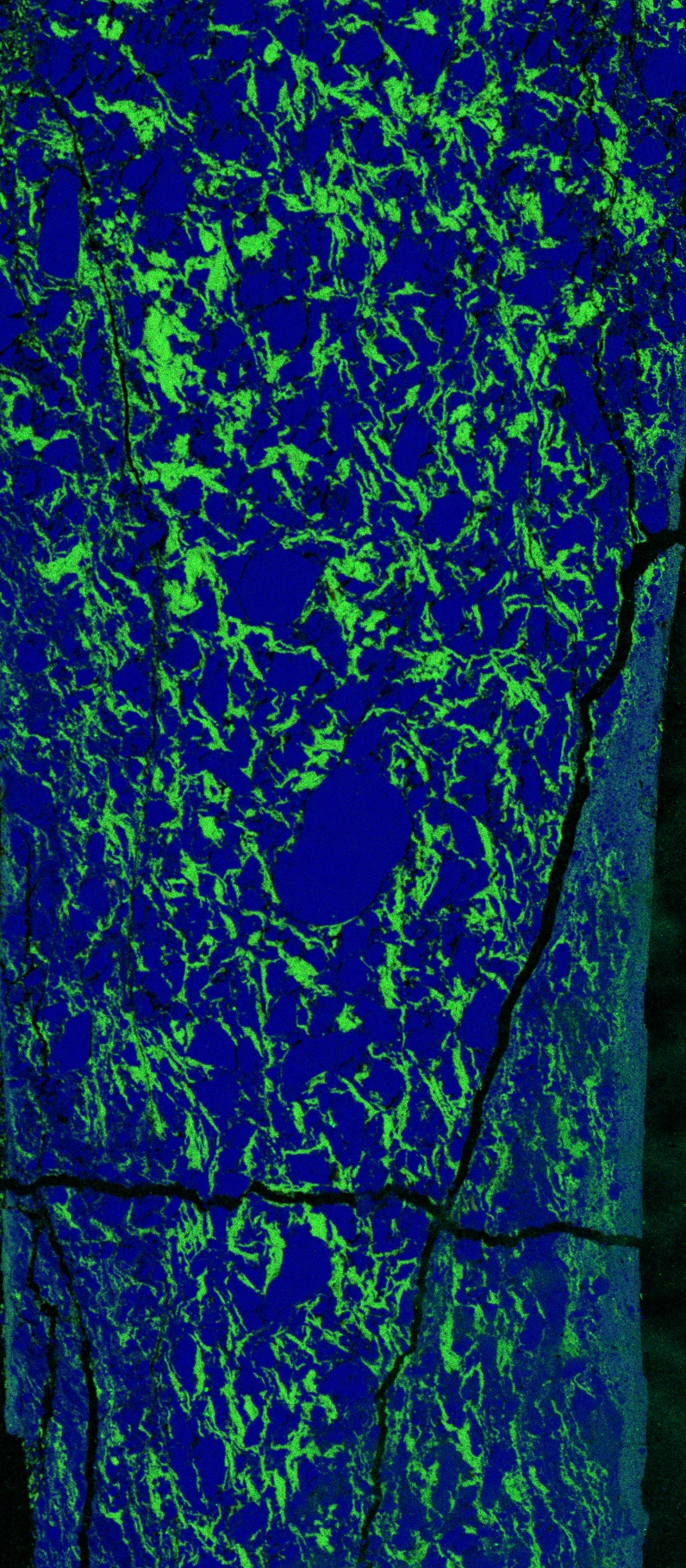
*

*Figure S8: RGB composite map of Si (blue), Al (green) and K (red) of sample u194, sheared at 0.3 μm/s, the starting microstructure. Data used in analysis of orientation of muscovite connected regions.*

*Resolution is 0.5 μm per pixel, map is 1400x3200 pixels.*

*
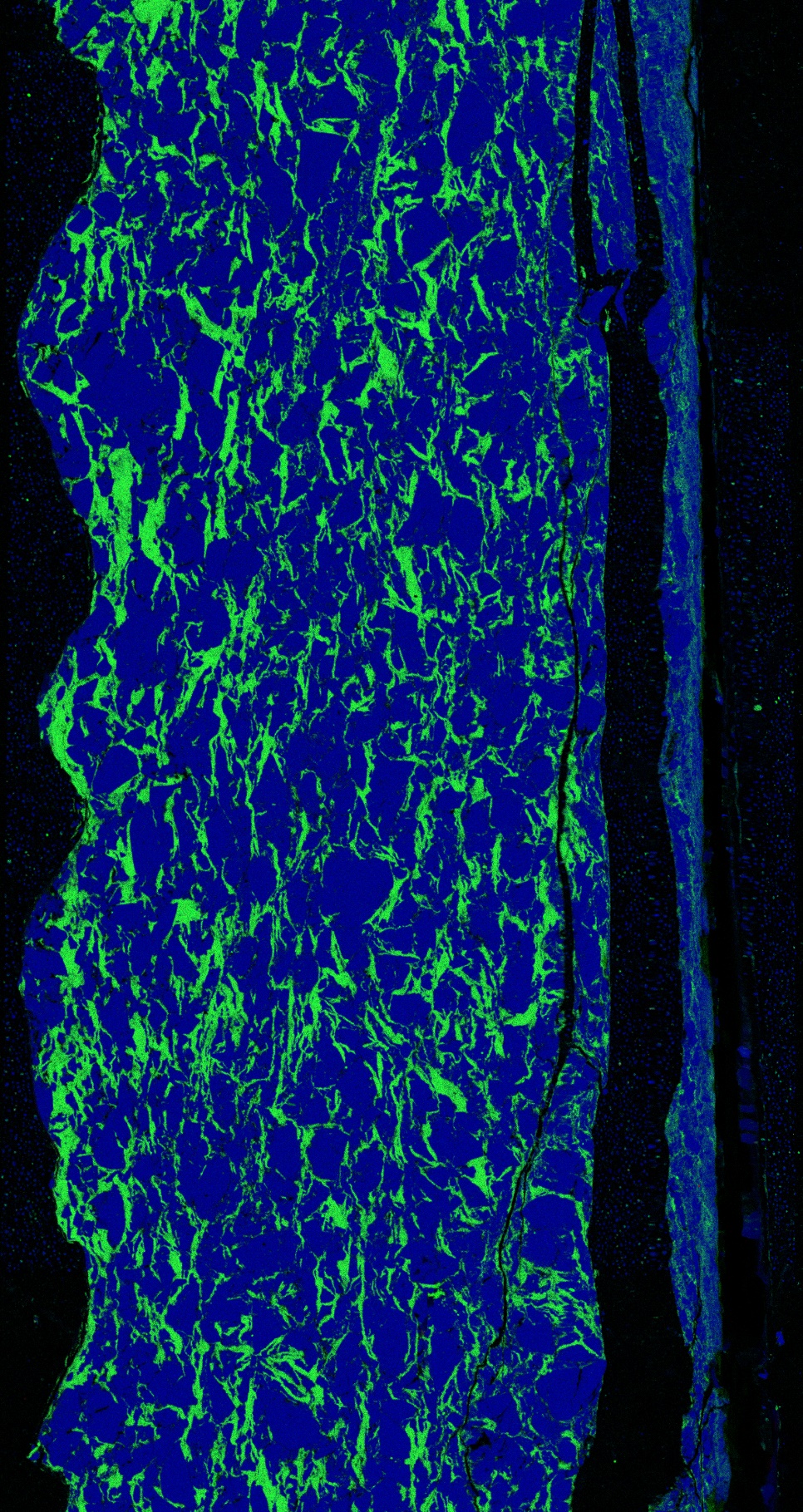
*

*Figure S9: RGB composite map of Si (blue), Al (green) and K (red) of sample u195, sheared at 0.03 μm/s, the starting microstructure. Data used in analysis of orientation of muscovite connected regions.*

*Resolution is 0.5 μm per pixel, map is 1700x3200 pixels.*

*Supplemental section 5): Results from an experiment on a stack of single crystals of muscovite, sheared at 0.03 μm/s, T=600 ºC and Pf of 120 MPa.*

*
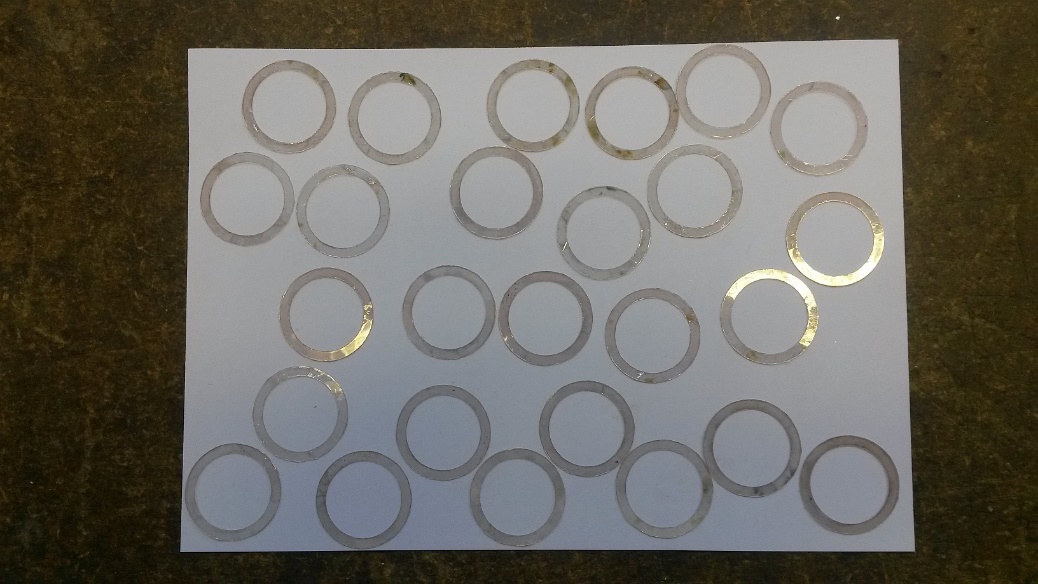

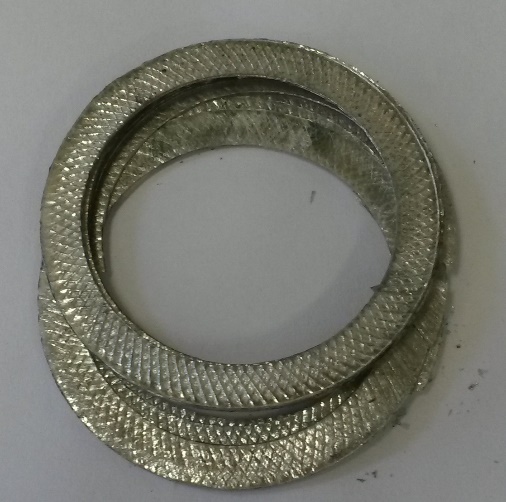
*

*Figure S10: Pictures of muscovite single sheet rings before (left) and after (right) the experiment.*

**

*Figure S11: Steady state shear stress as a function of effective normal stress for muscovite single crystals sheared at 0.03 μm/s, T=600 ºC and Pf of 120 MPa. Data corrected for seal friction shown in red. Slope yield the internal coefficient of friction which is about 0.08.*

*Supplemental section 6): Values for the input parameters for the two microphyiscal models shown in Figure 3.*

*Table S2: Input parameters used in the DH model, dissolution-controlled pressure solution.*

| **Parameter** | **Value (ref)** |
| --- | --- |
| Grain boundary (phyllosilicate) friction | 0.6 (den Hartog et al., 2013 |
| Dissolution velocity | 5.12x10^-9^ m/s (Tester et al., 1994) |
| Shape exponent *n* | 0.3 (den Hartog & Spiers, 2014) |
| Shape factor *k_f_* | 0.2 (den Hartog & Spiers, 2014) |
| Gouge layer thickness | 750 μm (Table 1) |

*Table S3: Input parameters used in the BNS model. Note that here both dissolution and diffusion contribute to the stress (diffusion does not become rate-controlling until a grain size of ~ 50 μm under these conditions)*

| **Parameter** | **Value (ref)** |
| --- | --- |
| Grain boundary (phyllosilicate) friction | 0.3 (Niemeijer & Spiers, 2005) |
| Dissolution velocity | 5.12x10^-9^ m/s (Tester et al., 1994) |
| Aspect ratio soluble grains | 4 (Bos & Spiers, 2002) |
| Active proportion foliation | 0.75 (Bos & Spiers, 2002) |
| Gouge layer thickness | 750 μm (Table 1) |
| Angle α steep part of foliation | 30 (Bos & Spiers, 2002) |
| Exponent *m, n* | 20 (Niemeijer & Spiers, 2005) |
| Diffusion coefficient | 9.69x10^-9^ m^2^/s (Gratier & Guiguet, 1986) |
| Solubility at 500 ºC | 0.002094904 m^3^/m^3^ (Rimstidt, 1997) |
| Fluid film thickness | 100 nm (Tada et al., 1987) |
